# Highly‐Time‐Resolved Measurements of Tryptophan's Distribution and Homeostatic Regulation in Live, Intact Animals

**DOI:** 10.1002/advs.77595

**Published:** 2026-10-15

**Authors:** Yuyang Wu, Chelsea N. Brown, Zeki Duman, Rose Mery Bakestani, Carl M. Kirkpatrick, Joao Hespanha, Tod Kippin, Kevin W. Plaxco

**Affiliations:** ^1^ Department of Chemistry and Biochemistry University of California Santa Barbara Santa Barbara California USA; ^2^ Institute of Collaborative Biotechnology University of California Santa Barbara Santa Barbara California USA; ^3^ Department of Electrical and Computer Engineering University of California Santa Barbara Santa Barbara California USA; ^4^ Monash Institute of Pharmaceutical Sciences Monash University Melbourne Australia; ^5^ Department of Psychological and Brain Sciences University of California Santa Barbara Santa Barbara California USA; ^6^ Neuroscience Research Institute University of California Santa Barbara Santa Barbara California USA; ^7^ Biological Engineering Graduate Program University of California Santa Barbara Santa Barbara California USA

**Keywords:** biosensor, EAB sensor, electrochemical aptamer‐based sensor, LM10, metabolomics, TDO inhibitor

## Abstract

Current theories regarding tryptophan's homeostatic regulation, which are derived from in vitro, post‐mortem, and cell‐culture studies, lack any direct evidence supporting their occurrence in living subjects. In response, here we have used 20 s resolution, multi‐hour plasma measurements to confirm, for the first time, that the cooperativity and tryptophan‐induced stabilization of tryptophan 2,3‐dioxygenase (TDO) seen in vitro and in cell culture hold in live, intact animals. For example, a model coupling these regulatory mechanisms with a constant tryptophan production rate from tissue reservoirs describes our in vivo measurements quantitatively, and provides high‐precision estimates of tryptophan flux and tryptophan‐induced TDO up‐regulation in individual subjects. In bridging a longstanding gap between ex vivo, post‐mortem, and cell culture investigations and the physiology of live, intact animals, an advance that had previously only been reported for glucose, our results highlight the transformative power of applying only‐recently‐available, seconds‐resolved metabolite measurements to the study of metabolic regulation.

## Graphical abstract

Using a novel, aptamer‐based biosensor, here we have performed 20‐s‐resolved, in situ measurements of plasma‐free tryptophan in the jugular veins of live rats (blue). This unprecedented time‐resolution provides insights into the regulation of tryptophan metabolism in live, intact animals. For example, due to small, tryptophan‐induced increases in the activity of hepatic tryptophan dioxygenase (modeled in green), repeated challenges with intravenous tryptophan accelerate the amino acid's elimination from the plasma (compartmental modeling and half‐lives shown in red).

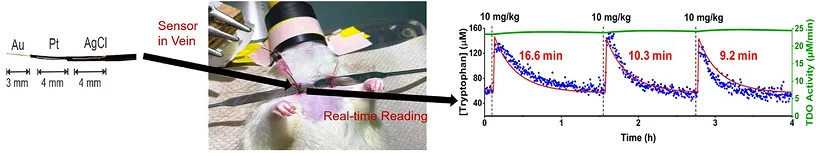

## 1 Introduction

While glucose is the most obvious example [1] of a plasma metabolite that is under strict homeostatic control, the plasma concentrations of many amino acids are similarly tightly regulated, no doubt due to the essential roles they play in protein synthesis, energy metabolism, nitrogen balance, and neurochemistry [2, 3, 4, 5]. Despite the importance of amino acid homeostasis, however, our understanding of the mechanisms by which it is acutely (i.e., over minutes) regulated remains far more limited than our understanding of blood sugar control. This is due to the cumbersome, poorly‐time‐resolved nature of prior methods of measuring the plasma concentrations of any metabolites other than glucose, all of which relied on conventional blood sampling and benchtop analytical techniques (e.g., chromatography‐mass spectrometry, enzyme assays, immunoassays). Until recently, for example, the best time‐resolved studies of phenylalanine metabolic kinetics in the rat used ex vivo measurements with sampling intervals of 15 min or longer [6, 7], producing resolution so poorly matched to the 3 to 9 min plasma clearance half‐life of this amino acid in this animal [8] that it is completely inadequate to test theories of the amino acid's homeostatic control. Fortunately, however, recent years have seen the development of a new technology, electrochemical aptamer‐based (EAB) sensors, able to perform seconds‐resolved, real‐time measurements of specific molecules in situ in the veins, brains, and solid peripheral tissues [8, 9, 10, 11, 12] that, unlike other generalizable in vivo sensor approaches [13, 14, 15], does not rely on the chemical transformation of its targets, and thus is applicable to the measurement of a wide range of molecular analytes. With these attributes, the EAB platform provides a unique means of testing theories of metabolic regulation in situ in live, intact animals.

In this paper, we have used 20 s resolution, in vivo EAB sensor measurements to explore regulation of the amino acid tryptophan, an essential amino acid (i.e., animals obtain it solely from the diet) that serves as a critical building block of proteins, cofactors, neurotransmitters, and neuromodulators [16]. Given these important roles, it is perhaps not surprising that plasma tryptophan is under tight homeostatic control. For example, a recent meta‐analysis that included more than 8000 fasted adult humans found that plasma tryptophan concentrations vary with a standard deviation of just 20% [17]. It is likewise not surprising that the dysregulation of tryptophan homeostasis contributes to a range of illnesses. A striking example is hypertryptophanemia, an inborn metabolic disorder in which chronically elevated plasma tryptophan concentrations lead to neurological and developmental anomalies [9]. This said, even modest deviations in plasma tryptophan are associated with disease; alterations in plasma tryptophan concentration and its ratio to total amino acid concentrations have been linked to obesity and increased risks of cardiovascular disease and epilepsy [18, 19]. Finally, dietary tryptophan intake is inversely correlated with self‐reported depression and positively associated with sleep duration [20], perhaps due to the roles that serotonin and melatonin play in regulating mood and wakefulness.

Previous studies suggest that the hepatic enzyme tryptophan 2,3‐dioxygenase (TDO) is responsible for 90% to 95% of the amino acid's degradation [21, 22, 23, 24], with regulation of this enzyme likely serving as the nexus for tryptophan homeostasis. Specifically, two of the enzyme's attributes are hypothesized to play important roles in regulating plasma free tryptophan concentrations. First, TDO has been shown to be cooperative in vitro [25, 26], with estimates of the rat enzyme's Hill coefficient ranging from 1.4 to 2.4. If this also holds in vivo, it would ensure that the enzyme's activity is low at normal physiological concentrations of tryptophan, but increases dramatically at higher concentrations. Second, tryptophan is thought to increase hepatic TDO activity. For example, in 1975, Badawy et al. reported that the tryptophan dioxygenase activity in the excised livers of rats sacrificed 3 to 5 h after a 50 mg/kg intraperitoneal tryptophan challenge is 2 to 9‐fold higher than that found in the livers of unchallenged control animals [27]. Similarly, Knox has reported that TDO activity increases 5‐fold in perfused, excised rat livers following a 30 min, 50 mg/kg tryptophan challenge [28]. Murine cell culture experiments paralleling these post‐mortem studies indicate that the effect arises due to a tryptophan‐induced slowing of the rate with which TDO is degraded [29]. Working in concert with the production of tryptophan from tissue reservoirs [16], the cooperativity of TDO and its tryptophan‐dependent half‐life are thought to be the dominant regulators of acute tryptophan homeostasis in mammals (Figure 1A).

**FIGURE 1:**
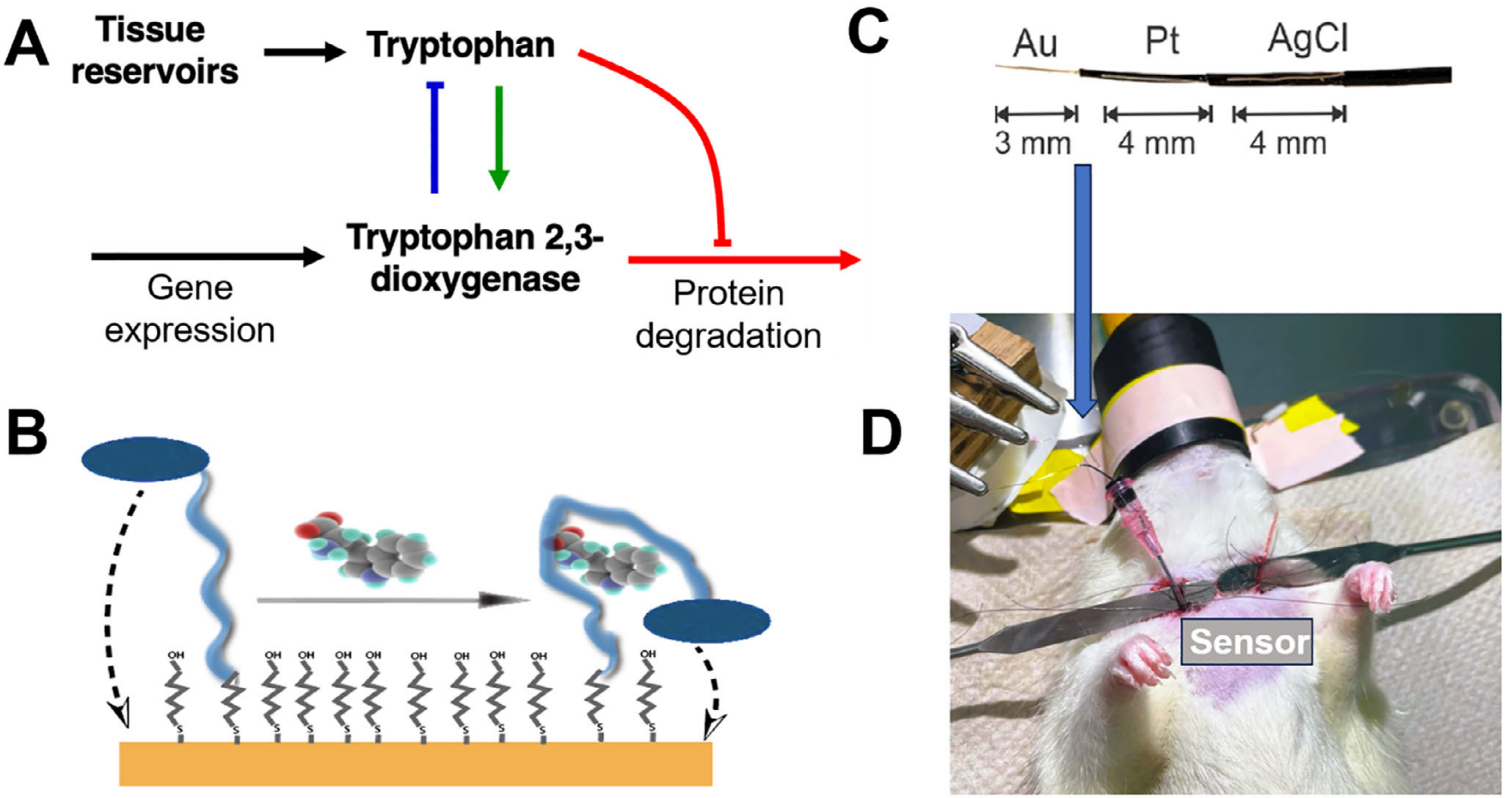
(A) Taken together, 5 decades of in vitro biophysical measurements, cell culture studies, and postmortem investigations of rat livers suggest that, in a fasted animal, plasma tryptophan concentrations are maintained by the interplay between the constant release of tryptophan from tissue reservoirs and its subsequent degradation by the hepatic enzyme tryptophan 2,3‐dioxygenase (blue bar). Specifically, these studies suggest that tryptophan homeostasis is regulated via tryptophan 2,3‐dioxygenase's positive allosteric cooperativity (green arrow) and inhibition of the enzyme's degradation rate by tryptophan (red bar). Here we use 20 s resolution, multi‐hour measurements of plasma‐free tryptophan collected in live, intact rats to access quantitatively the completeness of this vision of tryptophan homeostasis. (B) We performed these measurements using electrochemical, aptamer‐based (EAB) sensors, a technology that employs the binding‐induced folding of an electrode‐bound, redox‐reporter‐modified aptamer to generate an electrochemical output [34]. (C) For intravenous measurements, we fabricate our sensors on a 200‐µm‐diameter gold electrode paired with 127‐µm‐diameter counter (platinum) and reference (silver/silver chloride) electrodes. (D) The resulting bundle is inserted via a 22‐gauge catheter into the jugular vein of an anesthetized rat. (E, F) The resulting in vivo measurements achieve 20 s temporal resolution and good accuracy. Shown, for example, are measurements collected before and after intravenous tryptophan challenges in two rats at the indicated doses. To validate these in vivo measurements, we collected blood draws and performed ex vivo measurements using a commercial tryptophan assay. The free tryptophan concentrations thus determined (26, 294, 35, and 144 µM; denoted by “X”s) generally match those produced by the in vivo sensor (28, 296, 51, and 141 µM, respectively). The red curves are fits to a first‐order elimination, constant production rate model (Equation 1).

While in vitro, postmortem, and cell culture studies paint a consistent picture of the mechanisms underlying the regulation of acute tryptophan homeostasis, whether this picture accurately describes the physiology of intact animals remains in entirely unknown, primarily because of the poor time resolution of prior methods of measuring in vivo tryptophan concentrations [22, 30, 31, 32]. For example, the only prior literature description of the kinetics with which plasma tryptophan returns to homeostasis in rats, which employed a 1 h blood sampling interval, reported a 1.6 h plasma half‐life [33]. Given that this is an order of magnitude slower than the half‐life of the related amino acid phenylalanine [6], it likely reflects the time resolution of the measurements rather than the actual timescale of tryptophan regulation. Here, in contrast, we have used an in vivo EAB sensor [34] to measure plasma‐free tryptophan concentrations 180 times an hour over multi‐hour measurement runs conducted in situ in the rat jugular.

EAB sensors are comprised of a redox‐reporter‐modified, tryptophan‐binding aptamer immobilized on the surface of a gold electrode (Figure 1B,C). Upon binding tryptophan, this undergoes a conformational change that produces a measurable electrochemical signal. This conformation‐linked signal transduction mechanism renders the sensor single‐step, rapidly reversible, and selective enough for use in the complex environments found in vivo, thus enabling the high temporal collection of concentration measurements in the living body [8, 9, 10, 11, 12]. In providing this, EAB sensors offer a unique advantage over existing methods of monitoring amino acid metabolism in live subjects. For example, while microdialysis followed by HPLC or LC–MS/MS is highly specific and can simultaneously measure both tryptophan and its metabolites, the cumbersome nature of the approach typically limits its time resolution to tens of minutes per fraction and, due to incomplete recovery, the approach can be difficult to render fully quantitative [35]. Similarly, while stable‐isotope tracing provides a quantitative means of estimating metabolic flux, it provides little information regarding plasma half‐lives [36]. Finally, while fast‐scan voltammetry has been used to measure tryptophan in cell culture [37], the approach has not yet been adapted to work in the bodies of live animals; indeed, as it relies on direct tryptophan oxidation near +1 V (vs. Ag/AgCl), it is likely vulnerable to interference from other electroactive species present in vivo.

## 2 Results

### 2.1 In Vivo Sensor Validation

Our tryptophan‐detecting EAB sensors support the reproducible, specific, and stable measurement of tryptophan in body‐temperature whole blood. To see this, we collected fresh blood from a fasted rat, heparinized the blood, placed this in a 37°C water bath, and performed titrations to produce calibration curves. Between independently fabricated sensors (*n* = 7), these exhibit a relative standard deviation 3.9 to 6.1% (average 5.3%) across concentrations ranging from 20 to 1000 µM (Figure 2A). To correct for the drift inevitably seen under such challenging conditions, we employed ratiometric kinetic differential measurements [38], which reduced the mean signal drift to less than 5% after 15 h of continuous measurements (Figure 2B). To ascertain the specificity of our sensors, we challenged them in room temperature PBS buffer against the other aromatic amino acids, the key tryptophan metabolites, and a number of other potential interferents, each at 1 mM (Figure 2C). Even at this supraphysiological concentration, none of the interferents cased even a 10% change in signal, which is an order of magnitude smaller than the 110% signal change that 1 mM tryptophan produces.

**FIGURE 2:**
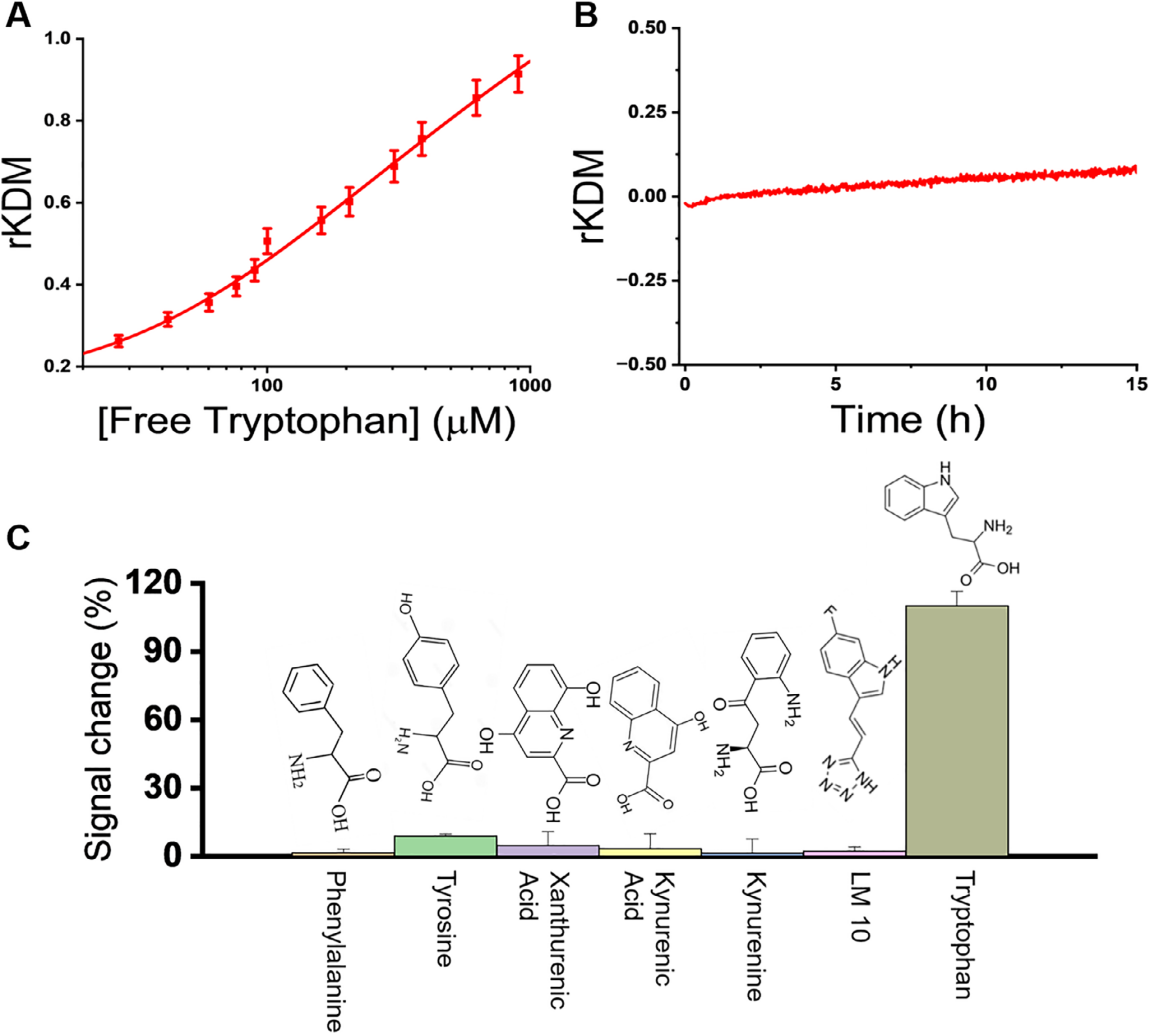
(A) Shown is a calibration curve (including standard deviations obtained from independently fabricated sensors; *n* = 7), with an RSD of approximately 3.9%–6.1% and an average RSD of approximately 5.3% across the tested range. (B) Using rKDM [38] to correct signal drift, the sensor  drifts less than 5% after 15 h of continuous measurement in undiluted, 37°C whole blood. (C) The tryptophan sensor is specific: when tested against aromatic amino acids, tryptophan metabolites and analogues at 1 mM in room temperature PBS buffer we see less than 10% signal change, which is small compared to the 110% signal change seen for tryptophan at 1 mM.

The tryptophan‐detecting EAB sensors produce accurate and precise, 20‐s‐resolved concentration measurements of plasma‐free tryptophan in situ in the bodies of live rats. To see this, we first placed a sensor in the right jugular of an anesthetized animal and collected 48 min of baseline measurements (Figure 3A). Over this period, the free tryptophan concentration varied from 17 to 32 µM. While collecting these baseline measurements, we also performed a blood draw from the animal, which we used to confirm the free tryptophan concentration using a benchtop enzymatic assay. The 26 µM concentration thus obtained closely matched the 28 µM value returned by the in vivo sensor at the same time point. Next, we challenged the animal with a 50 mg/kg intravenous tryptophan bolus delivered over 3 min via the other jugular. In response, plasma‐free tryptophan rapidly rose to 783 µM, after which it returned to basal levels over the course of a few tens of minutes. At approximately the half‐way point on the return to baseline, we collected a second blood sample for benchtop analysis. At 294 µM and 296 µM, respectively, this ex vivo measurement and the simultaneously obtained, EAB‐derived measurement likewise matched closely. As our second in vivo experiment, we challenged an anesthetized animal with two sequential intravenous tryptophan boluses (Figure 3B). Prior to the first challenge, the free tryptophan concentration ranged from 46 to 56 µM. Then upon challenging the animal with 10 mg/kg intravenous tryptophan bolus delivered over 3 min, plasma free tryptophan concentration rapidly rose to 302 µM before returning to a 54 µM baseline. Upon a second challenge, a 5 mg/kg intravenous tryptophan bolus delivered over 3 min, the free tryptophan concentration rose to 207 µM before returning to a baseline of 52 µM.

**FIGURE 3:**
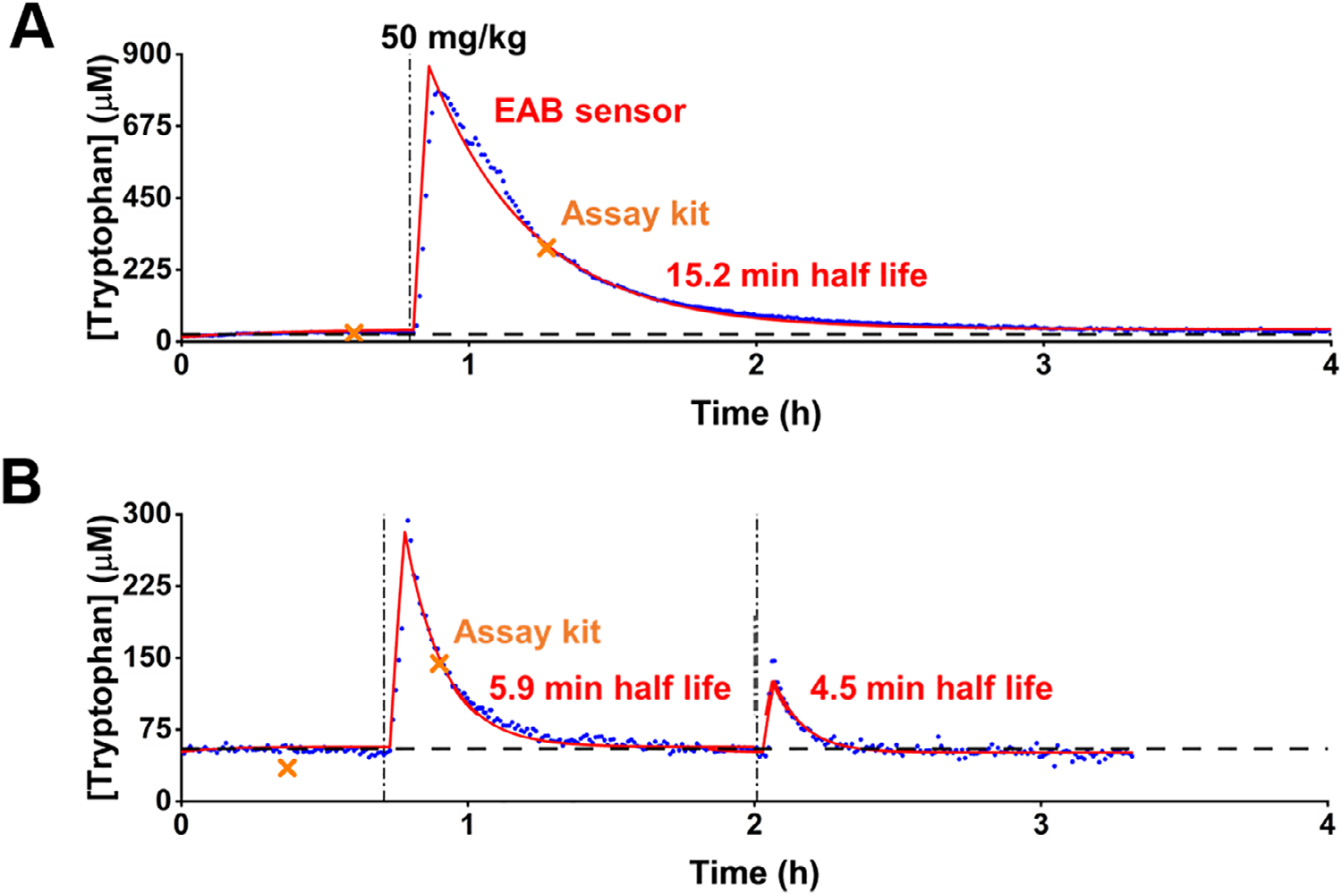
In vivo EAB sensors achieve 20 s temporal resolution and good accuracy. Shown, for example, are measurements collected before and after intravenous tryptophan challenges in anesthetized rats at the indicated doses. To validate these in vivo measurements, we collected blood draws and performed ex vivo measurements using a commercial tryptophan assay. The free tryptophan concentrations thus determined (26, 294, 35, and 144 µM; denoted by “X”s) generally match those produced by the in vivo sensor (28, 296, 51, and 141 µM, respectively). The red curves are fits to a first‐order elimination, constant production rate model (Equation 1).

The data quality and density provided by EAB sensors provide unique opportunities to explore detailed models of metabolic regulation in situ in living animals. As our first step in this direction, we fit our tryptophan measurements to a simple model including only first‐order elimination and a constant (i.e., time‐and‐tryptophan‐concentration‐independent) rate of tryptophan production from tissue reservoirs [16]:

(1) $$\frac{d\left[\mathrm{TRP}\right]}{dt}=\;\;\frac{\mu \left(t\right)+P_{\mathrm{TRP}}}{V_{D}}\;-\;k_{e}\left[\mathrm{TRP}\right]$$

Here [TRP] is the free plasma tryptophan concentration (in µM), *k_e_* is the tryptophan elimination rate constant (in min^−1^), *V_D_* (in L) is the tryptophan distribution volume, µ(t) is the experimentally‐defined rate with which exogenous tryptophan is infused (in µmol/min), and *P*_TRP_ is the endogenous tryptophan production rate (in µmol/min). From these fits we find that the estimated half‐life of the single intravenous challenge in our first animal (Figure 3A) was 15.22 ± 0.01 min (unless otherwise stated, all reported confidence intervals reflect estimated 95% confidence), and those of the sequential intravenous challenges we performed in our second animal (Figure 3B) were 5.88 ± 0.03 and 4.52 ± 0.07 min, respectively. These values are many‐fold faster than the 1.6 h plasma recovery half‐life previously reported for plasma tryptophan in the rat [33], a discrepancy that we attribute to the 180‐fold higher time resolution of our measurements, i.e., we believe that prior methods simply failed to capture the amino acid's true plasma half‐life.

### 2.2 TDO Cooperativity in Live, Intact Animals

In vitro, TDO is allosterically cooperative, with estimates [25, 26] of the rat enzyme's Hill coefficient ranging from 1.4 to 2.4 at physiological pH. If the enzyme is similarly cooperative in vivo, we should observe evidence of this when the plasma free tryptophan concentration significantly exceeds its Michaelis‐Menten constant, which in vitro studies have previously found to be either 221 or 360 µM at physiological pH [39, 40]. To test this, we challenged a rat with a 100 mg/kg intravenous tryptophan bolus delivered over 3 min, causing the amino acid's free plasma concentration to rise from a baseline of 46 µM to a peak of 638 µM before falling back to the same pre‐challenge baseline (Figure 4). We then fit the resulting time course to 2 models. The first is the simple, first‐order (i.e., lacking both enzyme saturability and cooperativity) model described by Equation (1). The second, a cooperative Michaelis‐Menten model, captures the in vitro‐observed cooperativity and saturability of the TDO enzyme. This model is given by:

(2) $$\frac{d\left[\mathrm{TRP}\right]}{dt}=\frac{\mu \left(t\right)+P_{\mathrm{TRP}}}{V_{D}}\;\;-\;\frac{V_{max}{\left[\mathrm{TRP}\right]}^{n_{H}}}{K_{m}^{n_{H}}+{\left[\mathrm{TRP}\right]}^{n_{H}}}$$

where *V_max_*, is the maximum velocity of TDO, *K_m_*, is the enzyme's Michaelis‐Menten constant, *n*_H_ is the Hill coefficient describing its cooperativity, and the other parameters are as defined for Equation (1). To better constrain this otherwise rather complex model (i.e., to reduce it from 5 fitted parameters to 4, allowing our model to converge on well‐constrained parameter estimates), we fixed *n*_H_ at 2, a value derived from prior in vitro characterization of the rat‐liver enzyme [25, 26].

**FIGURE 4:**
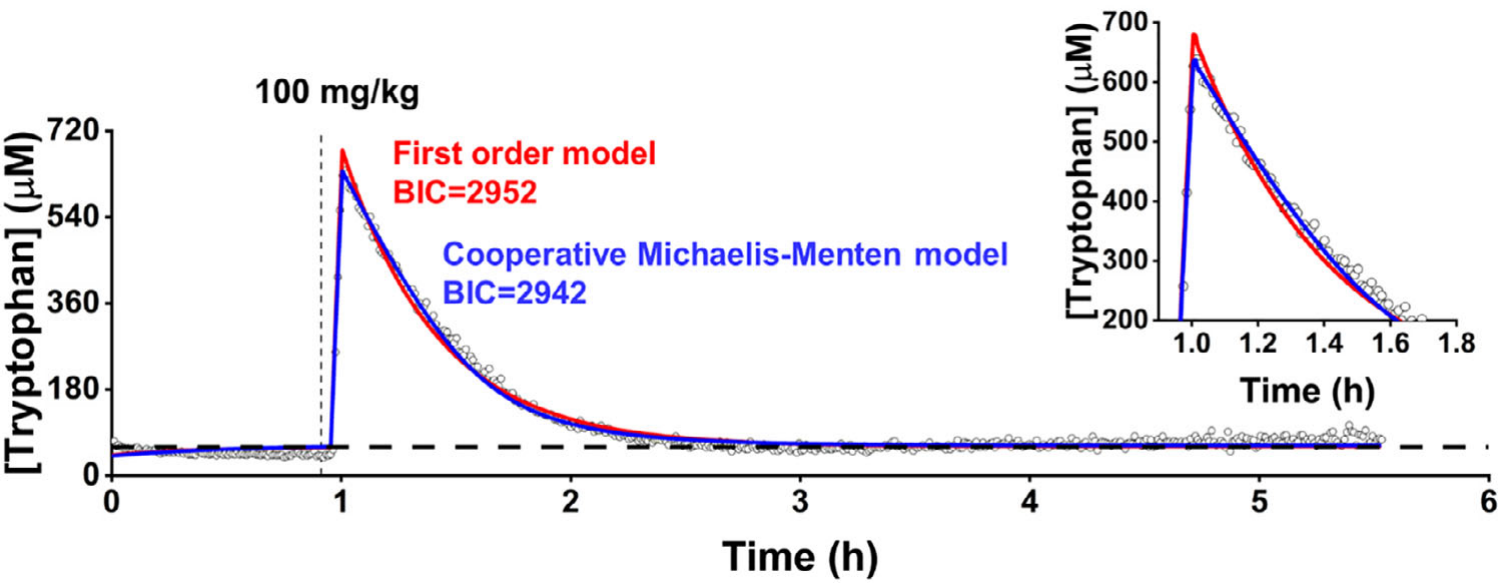
The cooperativity reported for the enzyme TDO in vitro also occurs in vivo. Applying a cooperative model (*n_H_* = 2) to the tryptophan elimination we see in vivo, for example, produes an estimate the enzyme's Michaelis‐Menten constant that, at 231 µM, falls between the 221 and 360 µM estimates previously reported for the rat liver enzyme in vitro [39, 40]. Inset is a zoom‐in around peak concentrations to illustrate the improved fit of the cooperative model relative to a non‐cooperative model. For example, within 30 min of C_max_ the root‐mean‐squared residuals fall from 16 µM for the first‐order model to 9 µM for the cooperative model (Figure S1). Consistent with this, the Bayesian Information Criterion (BIC) indicates the cooperative model is very strongly favored over the first‐order model. Note that it would not be possible to discriminate between the two models without the 20 s resolution in vivo measurements that EAB sensors uniquely provide.

The high temporal resolution measurements in vivo EAB sensors produce provide the first evidence that the cooperativity observed for TDO in vitro also holds in intact, live animals (Figure 4). For example, when we fit our 100 mg/kg challenge data to the cooperative TDO model, we recover a *K_m_* of 231 ± 1 µM, which falls within the 221 to 360 µM range previously reported for the rat enzyme in vitro [39, 40]. Likewise, the fit residuals seen at higher concentrations (i.e., for the 30 min after peak concentration) fall from a root‐mean‐squared value of 16 µM for the first‐order model to 9 µM for the cooperative model (Figure S1). To show that, in the face of the increased number of parameters in the latter model, this improvement in fit quality is statistically significant, we employed the Bayesian information criterion (BIC), a model selection analytic that evaluates which among a set of competing models is the most appropriate to describe a given data set by weighing goodness of fit while penalizing model complexity. Following Raftery's guidelines [41], a difference in BIC between the two models of 2 or more constitutes statistically significant evidence in favor of the model with the smaller BIC. For this 100 mg/kg challenge, the BIC of the cooperative model is, at 2942, 10 less than the 2952 of the first‐order model, rendering the former the strongly preferred description of this system. We note that, given the rather subtle differences in the fits, it would have not been possible to achieve this statistically significant demonstration that the cooperativity seen for TDO in vitro holds quantitatively in live animals without the hundreds of measurements per hour that EAB sensors uniquely provide.

### 2.3 Tryptophan‐Induced Increases TDO Lifetime in Live, Intact Animals

The high temporal resolution of our in vivo EAB sensors also provides the first evidence that, as expected given the tryptophan‐induced increases in hepatic TDO activity previously seen in post‐mortem [32, 42] and cell culture [43] studies, tryptophan challenges accelerate the elimination of plasma tryptophan in live, intact animals. For example, when we subjected an anesthetized rat to sequential 10 and 5 mg/kg tryptophan challenges, the return‐to‐homeostasis half‐life fell from 5.88 ± 0.03 min for the first challenge to 4.52 ± 0.07 min for the second (Figure 5A). To explore this further, we next challenged two rats with repeated 10 mg/kg challenges. Starting from a 57 µM basal free tryptophan concentration in one animal, we observed peak concentrations of 130 to 144 µM after each challenge. In each case, the free tryptophan concentration then returned to the same pre‐challenge baseline concentration (estimated post‐challenge baselines 54–63 µM), but it did so with half‐lives of 16.7 ± 0.1 min, 10.4 ± 0.1 min, 9.22 ± 0.08 min (Figure 5B), respectively. The second rat started at a basal free tryptophan concentration of 27 µM. After 4 sequential tryptophan challenges, this rose to peak concentrations of 109 to 123 µM (Figure 5C) before, each time, falling back towards the baseline concentrations with half‐lives of 11.33 ± 0.09 min, 7.42 ± 0.08 min, 8.81 ± 0.09 min, and 6.90 ± 0.09 min, respectively. (Due to animal welfare concerns that precluded keeping rats under anesthesia for more than 6 h, we were unable to wait until the tryptophan concentration returned fully to its baseline before delivering the next challenge dose.) Thus, for the 3 animals for which we performed sequential challenges, the elimination half‐life fell by 23% to 45% between the first and final challenges, as expected if tryptophan causes hepatic TDO levels to rise.

**FIGURE 5:**
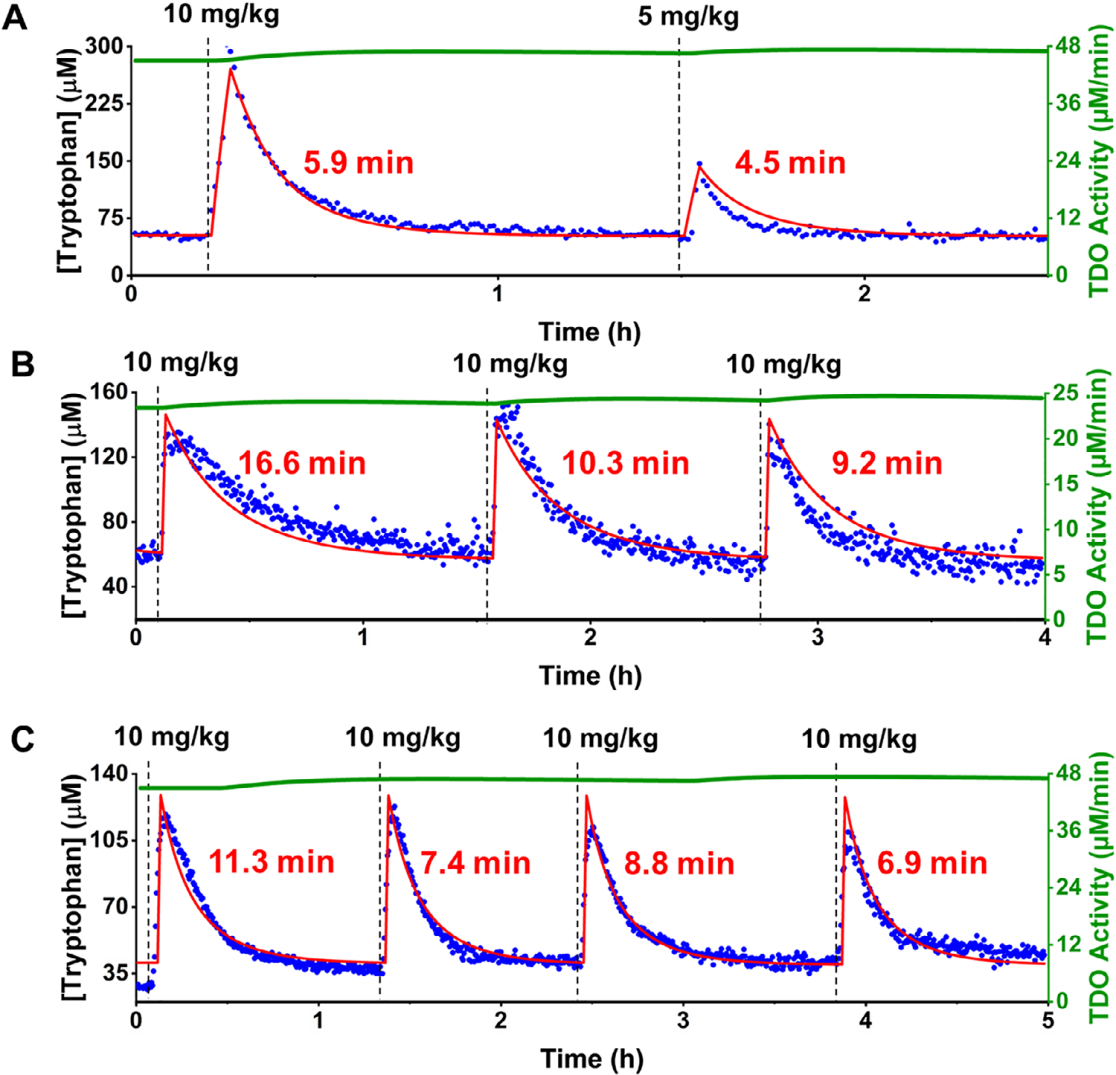
Postmortem, ex vivo studies of rat livers and studies of murine hepatic cell culture [29] indicate that TDO levels rise after a tryptophan challenge. We find this also holds in intact, live animals. To show this, we performed repeated tryptophan challenges, finding that, in these, the tryptophan elimination half‐life decreases with each subsequent challenge. A simple model that takes into consideration the first‐order dependence of the TDO half‐life on tryptophan concentration (red curves) accurately describes this effect. As expected (given the small size of these challenge doses), the model‐estimated increases in TDO activity that underlie the observed elimination rate acceleration, which are indicated (green curves), are only 5.5 to 8.5%. The indicated half‐lives (red font) are derived from first‐order model fits (not shown). Panel A is the same data presented in Figure 3B. TDO‐activity zoomed version of this figure is presented in Figure S2. We used a first‐order model (Equation 1) to extract the reported half‐lives of each individual challenge as our first modeling effort. Then we used the variable‐TDO model (Equation 2) to fit the entire time course and thus estimate changing TDO activity. Note that, in this model, the tryptophan elimination half‐live is no longer constant.

Tryptophan challenges in murine hepatic cell culture increase TDO activity by increasing the enzyme's half‐life, and thus its steady‐state concentration [26, 29, 44]. Assuming that this accounts for the accelerated tryptophan elimination we observed here, our data provide the first opportunity to identify and estimate the size of this effect in live, intact animals. To do so, we first expanded Equation (2) (which assumes the level of TDO activity is constant) to explicitly consider the time‐ and tryptophan‐dependence of TDO activity:

(3) $$\frac{d\left[\mathrm{TRP}\right]}{dt}=\frac{\mu \left(t\right)+P_{\mathrm{TRP}}}{V_{D}}\;-\;\mathrm{TDO}\left(t,\;\left[\mathrm{TRP}\right]\right)\frac{{\left[\mathrm{TRP}\right]}^{n_{H}}}{K_{m}^{n_{H}}+{\left[\mathrm{TRP}\right]}^{n_{H}}}$$

In this “variable‐TDO” model, the parameter TDO describes the amount of the enzyme in the animal as measured in “activity units,” the amount of enzyme that, at saturating substrate concentrations, would reduce plasma tryptophan by 1 µM/min. All other parameters are as defined above. Note that, whereas the parameters *P*_TRP_ and *V_D_* vary between animals, the parameters *K_m_* and *n*_H_ are intrinsic properties of TDO and thus should remain unchanged between animals. We thus fixed the latter two at 231 µM and 2, respectively, the values we determined from our 100 mg/kg challenge (Figure 4). Note, too, that the rate of exogenous tryptophan infusion, µ(t), is experimentally defined and is not a fitted parameter in our modeling.

In the variable‐TDO model, TDO activity depends on both time (due to the enzyme's relatively short half‐life) and the free tryptophan concentration (which extends this half‐life) [44], with the latter effect occurring through binding to a regulatory “exosite” that is distinct from the active site and that is not thought to be cooperative [43, 44, 45]. Given this, we model the time dependence of TDO activity as:

(4) $$\frac{d\left[\mathrm{TDO}\right]}{dt}=P_{\mathrm{TDO}}-\;k_{\mathrm{cat}}^{\mathrm{TDO}}\;\left[\mathrm{TDO}\right]\;\frac{K_{D}}{K_{D}+\left[\mathrm{TRP}\right]}$$

Here *K_D_* is the tryptophan affinity of TDO's exosite (µM), *P*_TDO_ and $k_{\mathrm{cat}}^{\mathrm{TDO}}$ are, respectively, the rate with which TDO is produced (µM/min^2^; i.e., the above‐defined “activity units”/min) and the first‐order rate constant for its degradation (min^−1^), both in the absence of tryptophan, and the other parameters are as defined above. To better constrain our model, we assumed that the half‐life of the rat enzyme in the absence of tryptophan matches the 82 min half‐life of the mouse enzyme in hepatic cell culture [29]; fit quality, however, is not particularly sensitive to the exact value we employ here (data not shown). We also assume that the half‐life of tryptophan‐bound TDO is effectively infinite (i.e., much longer than the timescale of our experiments). In support of this assumption, at 80 µM tryptophan the half‐life of TDO in cultured mouse cells is much greater than 8 h [29]. Finally, while the value of *K_D_* does not yet appear to have been determined for the rat protein, under the argument that meaningful regulation can only occur if this value is near the physiological tryptophan concentration, we constrained it to 100 µM; our fits are likewise not particularly sensitive to this parameter (data not shown). The variable‐TDO model thus contains only 3 fitted parameters (*V_D_, P*_TRP_, and *P*_TDO_), each of which varies between animals but is assumed to remain constant for any one individual over the duration of the experiment. All of the other parameters in our model, in contrast, are fixed.

Despite its relative simplicity, the variable‐TDO model of tryptophan homeostasis describes our 20 s resolved, multi‐hour plasma‐free tryptophan time courses quite well (Figure 5). For example, across our 3 multi‐challenge data sets, the mean absolute deviation between the model and our measurements ranges from 3.9 to 7.8 µM and the mean absolute relative deviation from 2.9 to 10.5% (Table 1). And while, by BIC, the varying‐TDO‐activity model is just as favored as the fixed‐TDO‐activity model for our dual challenge data set (BIC = 1884 and 1883, respectively), the variable‐TDO model is the strongly preferred description of our triple (5408 vs. 5447; note again that a difference as small as 2 is considered statistically significant evidence in favor of the model with the lower BIC [41]) and quadruple (5711 vs. 5728) challenge data sets (Figure S3). Here, too, it would not have been possible to discriminate between these two models with statistical significance without the hundreds of measurements per hour that EAB sensors provide.

**TABLE 1:** Animal characteristics, fitted kinetic parameters, goodness of fit metrics.

	Animal characteristics			Fitted parameters				Derived parameters		Fit metrics	
Figure No.	Feeding status^a^	Sex	Mass (g)	*V_D_* (L)	*P*_TRP_ (µmol/min)	*P*_TDO_ (µM/min^2^)	[LM10] (µM)	Flux_TRP_ (µmol/kg/h)	ΔTDO (%)^d^	MAD (µM)	MARD (%)
5a^b^	Fasted	F	321	0.065	0.14	14.9	—	27.0	5.5	6.6	9.4
5b^b^	Unfasted	M	571	0.315	0.42	7.3	—	44.7	5.1	7.8	10.5
5c^b^	Fasted	M	636	0.346	0.43	15.0	—	40.8	8.5	3.9	7.3
6a^c^	Unfasted	M	426	0.145	0.34	3.9	14	47.9	9.1	3.7	2.9
6b^c^	Unfasted	M	450	0.050	0.22	9.9	47	29.3	20.4	8.8	5.9
6c^c^	Unfasted	M	508	0.242	0.45	9.2	66	53.1	10.3	6.8	7.8

^a^ Unfasted = ad libitum food access; Fasted = without food for 24 h.

^b^ Values for the 1‐compartment, variable‐TDO model.

^c^ Values for the 2‐compartment, variable‐TDO model.

^d^ Estimated change in total TDO activity between the start of observations and the highest estimated TDO activity.

### 2.4 TDO Inhibitor‐Altered Tryptophan Kinetics in Live, Intact Animals

The ability to monitor metabolite concentrations with sub‐minute resolution provides opportunities to measure the kinetics with which drugs modulate metabolism (i.e., pharmacodynamics) with unprecedented precision. To illustrate this, we have also measured the extent to which and the speed with which basal tryptophan concentrations respond to LM10, a specific inhibitor of TDO that does not inhibit tryptophan degradation via its other, non‐hepatic pathways [46]. To do so, we challenged 3 animals with a single, 10 mg/kg intraperitoneal dose of the inhibitor (Figure 6). If dispersed through the total extra‐ and intra‐cellular fluid volume of the rat [47], this corresponds to concentrations of ∼65 µM, a value that, given the LM10's reported 24 to ≥50 µM inhibition constant in cultured mammalian cells [48], should reduce TDO activity but not abolish it. Consistent with this, within 30 to 40 min after LM10 dosing, the basal free tryptophan concentrations in the 3 animals rose by 15% to 40% and then remained at this new, higher level. (Note that, despite the inhibitor's structural similarity to tryptophan, the sensor does not appreciably respond to LM10; Figure 2C.) When, some 1.5 to 2.5 h after the LM10 dose, we challenged these rats with 10 mg/kg intravenous tryptophan, its plasma concentration rose before then returning to the new, higher basal level. As expected, the mean half‐life of this recovery was slightly slower (mean = 13.7 min, standard deviation = 7.4 min, *n* = 3) for the LM10‐treated animals than the mean “first‐challenge” half‐life seen for untreated animals given the same 10 mg/kg tryptophan challenge (11.3 min, standard deviation = 4.4 min, *n* = 3).

**FIGURE 6:**
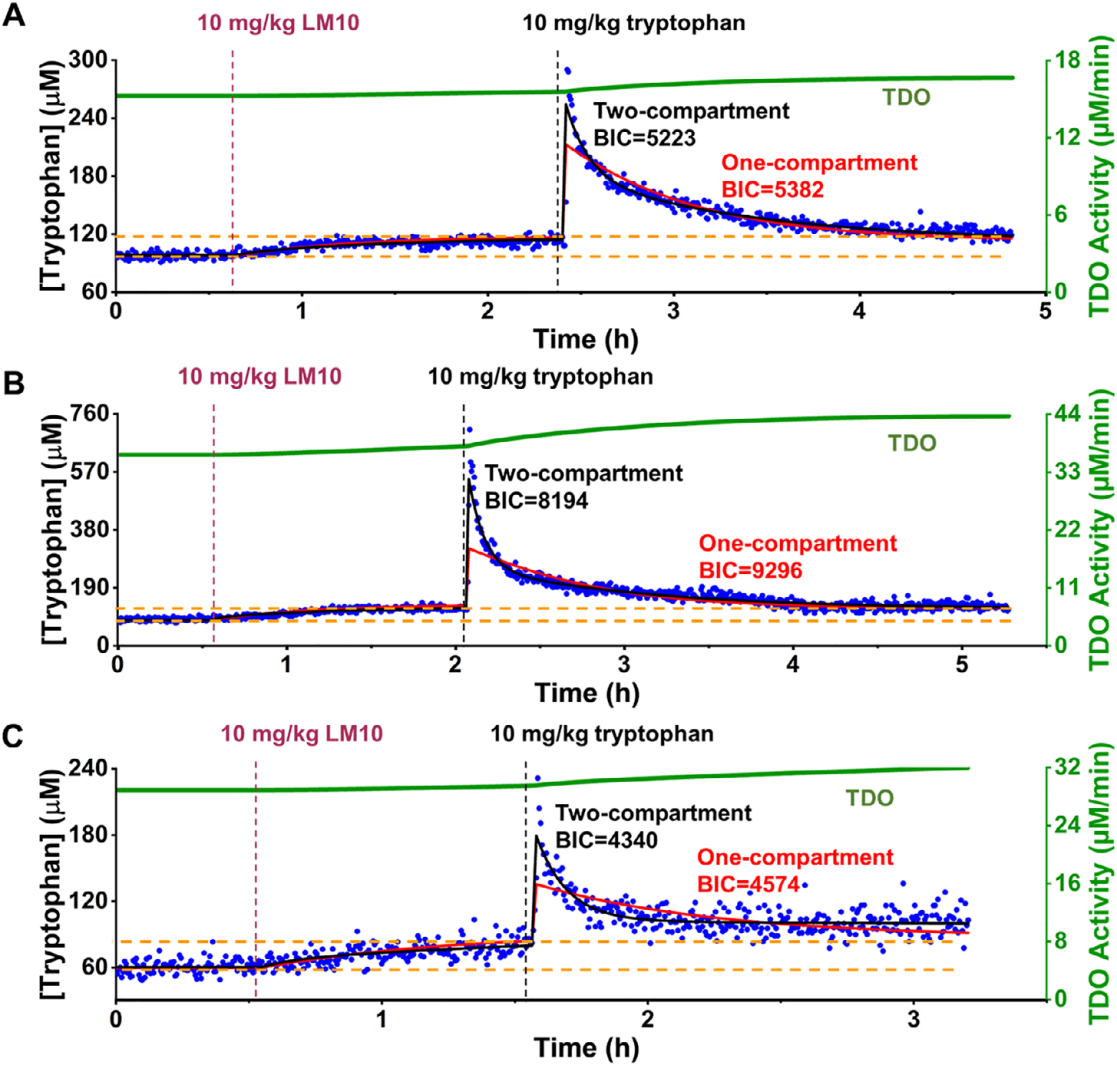
Prior studies have argued that TDO is responsible for 90% to 95% of tryptophan degradation. Consistent with this, we find that administration of the TDO‐specific inhibitor LM10 leads to 15% to 40% increases in free basal tryptophan concentrations (dashed horizontal lines indicate pre‐ and post‐LM10‐challenge baselines). After a subsequent intravenous tryptophan challenge, the concentration of the amino acid returns to the new, higher basal concentration. Adding LM10 inhibition to our variable‐TDO model of tryptophan homeostasis accurately captures the rise in basal concentrations (red curves), but it fails to capture the unusually high *C_max_* we observe upon subsequent tryptophan challenge. Adding a second, unobserved compartment to this model, however, leads to statistically significantly improved fits (black curves), suggesting that, in addition to inhibiting TDO, LM10 also causes the amino acid's normally rapid distribution into the tissues to slow enough that it becomes measurable with the 20 s resolution of our data. The model‐estimated increases in TDO activity (from the two‐compartment model) are also indicated (green curves). The rise in baseline is highlighted in Figure S4. Note that LM10 does not measurably cross‐react with our sensor (Figure 2C). TDO‐activity zoomed version of this figure is Figure S5.

The observation that TDO's cooperativity and tryptophan‐regulated lifetime are sufficient to account for tryptophan homeostasis in live rats also holds for the studies in which we employed the TDO inhibitor LM10. To see this, we add an additional term to Equation (3) describing the inhibition of TDO by LM10:

(7) $$\begin{array}{ccc} \frac{d\left[TRP\right]}{dt} & = & \frac{\mu \left(t\right)+P_{TRP}}{V_{D}}-TDO\left(t,\;\left[TRP\right]\right)\\ & & \times \frac{{\left[TRP\right]}^{n_{H}}}{K_{m}^{n_{H}}+{\left[TRP\right]}^{n_{H}}}\;\frac{K_{i}}{K_{i}+\left[LM10\right]} \end{array}$$

Here [LM10] is the fitted concentration of the inhibitor (in µM), *K_i_* is its inhibition constant (which, based on cell culture studies, we fixed at 40 µM), and the other parameters and their fixed values are as described above.

In combination with the tryptophan‐dependence of the TDO lifetime (Equation 4), this enhanced, variable‐TDO model provides an accurate description of the rise in plasma tryptophan concentrations seen following challenge with LM10 (red curves in Figure 6). In contrast, however, it does a relatively poor job of capturing the maximum plasma tryptophan concentrations, *C_max_*. We note too, however, that the *C_max_* we observed for our LM10‐treated animals is higher than those seen for untreated animals (Figure 5). Specifically, the mean *C_max_* seen after 10 mg/kg tryptophan challenges in LM10‐treated animals was 402 µM (standard deviation = 219 µM, *n* = 3), vs. the 184 µM (standard deviation = 83 µM, *n* = 3) mean *C_max_* seen following the first tryptophan challenge in untreated animals.

As judged by the excellent fits of first‐order models lacking a distribution phase, it appears that, in the absence of LM10, tryptophan's distribution phase is too rapid for even the 20 s resolution of our sensors to capture (Figure 4 and 5). In contrast, when we employ a variable‐TDO model that includes 2 compartments, the plasma and a second, “distribution” compartment, we find that, upon LM10 treatment, the distribution phase becomes kinetically distinguishable, explaining the much higher *C_max_* we observe in LM10‐treated animals. The expanded model employs Equation (4) to describe the time‐and‐tryptophan‐dependent evolution of TDO, a version of Equation (7) expanded to include transport to and from the new compartment,

(8) $$\begin{array}{ccc} \frac{d{\left[TRP\right]}_{P}}{dt} & = & \frac{\mu \left(t\right)+P_{TRP}\;}{V_{D}}\;-\;TDO\left(t,\;\left[TRP\right]\right)\;\frac{{\left[TRP\right]}_{P}^{n_{H}}}{K_{m}^{n_{H}}+{\left[TRP\right]}_{P}^{n_{H}}}\\ & & \times \frac{K_{i}}{K_{i}+\left[LM10\right]}+k_{DP}\left({\left[TRP\right]}_{P}-\;{\left[TRP\right]}_{D}\right) \end{array}$$

and a new equation describing the tryptophan concentration in that unobserved compartment.

(9) $$\frac{d{\left[TRP\right]}_{D}}{dt}=k_{PD}\left({\left[TRP\right]}_{D}-\;{\left[TRP\right]}_{P}\right)$$

Here [TRP]*_P_* and [TRP]*_D_* are the tryptophan concentrations in the plasma and the (unobserved) distribution compartment, *k_PD_* and *k_DP_* are the rate constants for transport from the former to the latter and vice versa, and the other parameters are as defined above. Using this model, we find that it provides a statistically significantly improved fit for the challenge data collected in LM10‐treated animals (Figure 6, black curves). Specifically, in all 3 cases the BIC of the 2‐compartment model is significantly smaller than that of the 1‐compartment model. In contrast, the 2‐compartment model is the preferred model for only 1 of the 3 multi‐challenge experiments we performed in the absence of LM10 (Figure S6), suggesting that LM10 is significantly slowing trytophan's distribution into the tissues.

## 3 Discussion

Prior theories regarding the metabolic homeostasis of tryptophan were based entirely on ex vivo [27, 28], postmortem [32, 42], and cell culture [29, 43] studies, leaving open the question as to whether the mechanisms they identified occur in live, intact animals at all, much less whether they constitute a largely complete description of the regulation of this essential amino acid. Here, however, we have used 20 s resolution, multi‐hour measurements of plasma free tryptophan and its response to intravenous challenges with exogenous tryptophan and a TDO‐specific metabolic inhibitor to, for the first time, test these theories in the context of fully functional, intact animals, with all of the physiological complexities this entails. From this, we find that models of tryptophan homeostasis encompassing only the cooperative nature and tryptophan‐induced stabilization of hepatic TDO capture both the kinetics of tryptophan clearance after exogenous tryptophan challenge, and the extent to which such clearance accelerates in response to repeated tryptophan challenges. This simple model is also sufficient to account for the increase in tryptophan baseline and the deceleration of tryptophan clearance in animals treated with the TDO‐specific inhibitor LM10. Together, these suggest that current theories of the regulation of tryptophan homeostasis, which were based solely on in vitro studies of the purified enzyme, post‐mortem studies of excised rat livers, and cell culture studies of hepatocytes, is sufficient to explain the homeostatic regulation of plasma tryptophan in intact, living animals, at least under the experimental conditions (i.e., anesthetized animals, few‐hour timescale) we have employed.

In addition to providing the first test of current understanding of the homeostatic regulation of any metabolite other than glucose in an intact, live animal, our studies also provide high‐precision estimates of the parameters that describe tryptophan metabolism in individual rats (Table 1). For example, while distribution volumes are a theoretical concept that does not necessarily correlate with the volume of any specific bodily compartment, the values we obtained for tryptophan are at least physiologically plausible. Specifically, the estimated distribution volumes of the non‐LM10‐treated rats are within a factor of 3 of the total fluid volume of a rat [47]. The model's estimated basal tryptophan production rates, which varied between 0.14 and 0.45 µmol/min over the animals we characterized, are likewise plausible. As expected, for example, the tryptophan production rate is reasonably well correlated (R = 0.85) with body mass, with a y‐intercept within error of zero (Figure 7). That is, larger animals produce tryptophan from tissue reservoirs more rapidly, presumably due to their larger muscle mass. The observed tryptophan production rates also compare closely with the production “fluxes” of similar amino acids in other mammals. To see this, we used each rat's measured body mass and estimated tryptophan production rate to calculate their total body tryptophan flux, the mean and standard deviation of which are 40.5 ± 10.4 µmol/kg/h. Although we have not found any independent measurements of this specific flux in any mammal, isotopic tracer studies in humans indicate that, irrespective of feeding status, the whole‐body fluxes of the related amino acids tyrosine and phenylalanine are, respectively, 28 or 35 µmol/kg/h and 40 or 46 µmol/kg/h [49, 50]. It thus appears that the tryptophan fluxes we have estimated are closely similar to the production rates of the other aromatic amino acids in another mammal. Prior studies also fail to provide any direct means of judging the reasonableness of the model's estimated TDO production rates. Nevertheless, the 5% to 20% increases in TDO activity that the model predicts (Table 1 and Figure 5 and 6) over the course of a few hours in response to 10 mg/kg tryptophan challenges are at least qualitatively consistent with prior reports of several‐fold increases in TDO activity many hours after 5‐fold larger challenges [23]. Finally, at 14 to 66 µM the estimated LM10 concentrations in the 3 animals that received the inhibitor are satisfactorily close to the expected 65 µM value obtained by distributing the 10 mg/kg dose throughout the total fluid volume of a rat. Especially given that, while our model assumes the drug's half‐life is far longer than the ∼3 h observation period of the relevant experiments, pharmacokinetic studies have found its plasma half‐life is only a ∼2 h in mice [51], which would push the average concentration below our rather approximate 65 µM estimate. Taken together, the physiological and pharmacological “reasonableness” of these parameter estimates provides further evidence of the completeness of our simple model of tryptophan homeostasis under the experimental conditions we have employed.

**FIGURE 7:**
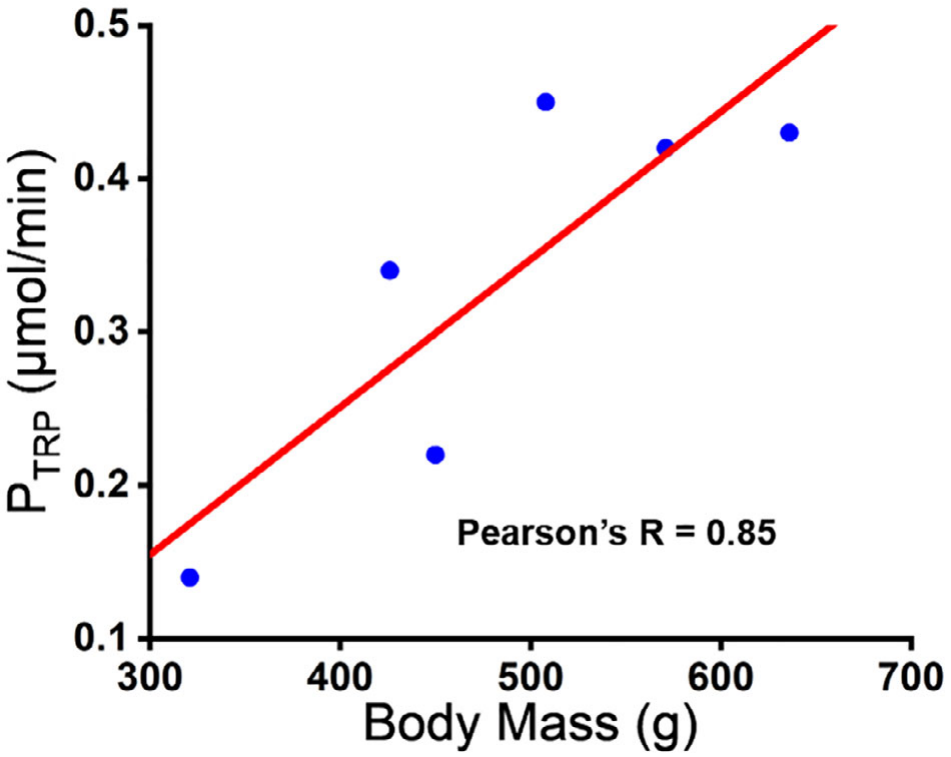
As expected, the tryptophan production rate is larger for larger animals, presumably due to their greater muscle mass. Specifically, the correlation between tryptophan production rates and body mass is given by *P_TRP_* = 0.000963(mass) – 0.13(±0.15), which achieves a Pearson's correlation coefficient of 0.85.

The unprecedentedly high temporal resolution measurements of plasma tryptophan EAB sensors produce also provide insights into the kinetics with which tryptophan is distributed from the plasma into the tissues. Specifically, our data suggests that the transport of the amino acid out of the plasma is remarkably rapid; despite the 20 s resolution of our measurements, we see no statistically significant evidence of a distribution phase in the absence of LM10 treatment (Figure 4 and 5). Specifically, the post‐challenge kinetics we observed in these animals are well fitted by single‐compartment models lacking any distribution phase, suggesting that the body is acting as a single, rapidly (relative to the 1 min infusion period) equilibrating compartment. This observation is consistent with recent measurements of the transport of tryptophan into HeLa cells, which equilibrates with a time constant of just ∼0.5 min [52]. In LM10‐treated animals, in contrast, the distribution phase becomes slow enough that our measurements easily capture it. Specifically, the kinetics we measured after tryptophan challenges in LM10‐treated animals are better described by a 2‐compartment model (as determined using BIC; Figure 6), with a distinct, easily detected distribution phase with a mean time constant of 4.3 ± 2.0 min (Figure S7). While speculative, we posit that this LM10‐induced deceleration could arise due to the inhibition of tryptophan transport into cells by this tryptophan analog.

Notwithstanding the accuracy of our model in describing the details of tryptophan homeostatic regulation and transport in our experimental setting, we recognize that the model we employed ignores other potentially important aspects of tryptophan metabolism and its regulation. For example, the extra‐hepatic enzyme indoleamine 2,3‐dioxygenase diverts tryptophan into a kynurenine pathway parallel to that initiated by hepatic TDO, and tryptophan hydroxylase diverts it into the synthesis of serotonin. Prior studies have shown, however, that TDO accounts for 90% to 95% of total tryptophan flux in both rats and humans [16, 18, 51]. The ability of our model (which, again, considers only the biophysical parameters of TDO) to accurately describe the tryptophan dynamics we observe despite ignoring these other pathways provides further evidence that their contributions to total tryptophan flux in the rat are small. Our model also ignores the known hormonal regulation of tryptophan metabolism. For more than 65 years, for example, it has been known that glucocorticoids increase hepatic TDO activity in live rats [53, 54, 55]. Similarly, the administration of high‐dose dexamethasone to cultured rat hepatocytes increases their TDO activity 7‐8‐fold, an effect that occurs via hormone‐induced increases in gene expression [56]. The timescale of this induction, however, is more than 5 h, suggesting that it is unlikely to contribute significantly to the acute tryptophan dynamics captured in our experiments. In contrast, insulin and glucagon are known to modulate the relative rates of protein synthesis and protein degradation in tissue reservoirs over timescales of a few tens of minutes [57]. Nevertheless, despite ignoring both these hormonal regulatory mechanisms and alternative metabolic pathways, our model describes the tryptophan dynamics we have measured in intact animals rather well, at least under the experimental conditions we have employed.

As illustrated here, the ability of EAB sensors to measure metabolite concentrations and their clearance kinetics with seconds‐scale resolution and multiple‐hour duration provides unique opportunities to test and refine our understanding of the homeostatic regulation of metabolites other than glucose in intact, live animals. That is, while here we have employed 20 s resolution measurements as a means of studying tryptophan homeostasis, the generality of the EAB platform suggests that it could also be used to explore the regulation of a much wider range of metabolites, provided the relevant aptamers are available. The demonstrated ability of EAB sensors to perform similarly well‐resolved, multi‐hour measurements in situ in other bodily compartments, including the solid peripheral tissues [58] and the brain [59], likewise suggests that this new technology may pave the way for significantly improved, systems‐level understanding of the inter‐organ transport of metabolites. Finally, the ability of EAB sensors to not just measure, but also control (i.e., closed‐loop feedback control) in vivo metabolite concentrations [59, 60, 61] would likely also be of significant value in the study of metabolic regulation. In short, EAB sensors offer a unique window into molecular physiology, its regulation, and its dysregulation in both health and disease.

## 4 Materials and Methods

### 4.1 Materials

Phosphate‐buffered saline (PBS) and salts were obtained from Santa Cruz Biotechnology (Dallas, TX), 7.5% sodium hypochlorite household bleach from Clorox (Oakland, CA), tryptophan from Thermo Fisher Scientific (Waltham, MA), LM10 from TargetMol Chemicals Inc. (Boston, MA), and cow blood from Hemostat Laboratory (Dixon, CA). Gold wire (0.2 mm diameter), platinum wire (0.005 in. diameter), and silver wire (0.005 in. diameter) were purchased from A‐M Systems (Sequim, WA). Polyolefin heat‐shrink tubing was purchased from McMaster‐Carr (Elmhurst, Illinois) and PTFE tubing (HS Sub‐Lite‐Wall, 0.02 in, black opaque) from Zeus (Branchburg Township, CA). 60/40 lead‐selenium solder was purchased from Digikey (Thief River Falls, MN). Gold‐plated pin connectors, platinum counter electrodes and Ag/AgCl (3 M KCl) reference electrodes were obtained from CH Instruments (Austin, TX). We obtained the thiol‐ and methylene‐blue (MB)‐modified aptamer from IDT (Newark, NJ) and used as received with the following structure:

5’‐HO‐(CH_2_)6‐S‐S‐(CH_2_)_6_‐O‐PO_2_O‐ CCG GTG GTG TAG TTC CGG CGT GGG GAA GG‐O‐CH_2_‐CH(CH_2_OH)‐NH‐C(O)‐(CH_2_)_2_‐MB‐3’

### 4.2 Sensor Preparation

In this work, we have employed a previously‐described EAB sensor against tryptophan [34], which we adapted to in vivo sensing as follows. We first insulated 5 cm gold wire with heat‐shrinkable Polyolefin tubing, leaving approximately 3 mm wire exposed as the working electrode. We soldered the other end to a gold‐plated pin connector with 60/40 lead‐selenium solder before deploying the electrode in vitro with a platinum wire counter and a commercial Ag/AgCl reference. For in vivo sensors, we bundled 10 cm gold, platinum, and silver wires that were individually insulated with PTFE heat‐shrink tubing, together in a staggered manner such that their exposed lengths were 3, 6, and 1 cm, respectively. We next immersed the bundled electrodes in household bleach to chlorinate the silver electrode before rinsing all three with distilled, de‐ionized water prior to electrochemical cleaning.

We converted these electrodes into in vivo EAB sensors using established protocols [59, 60, 61]. In brief, we first exposed the bare gold wire to 0.5 M NaOH and then used external counter and reference electrodes and a CHI 1040C Electrochemical Workstation (CH Instruments of Austin, TX) to perform electrochemical cleaning: cycling the potential 300 times between −1 and −1.6 V (all potentials vs. Ag/AgCl) at 1 V/s to remove any residual contaminants on the electrode surface. Afterwards, we rinsed the electrode and performed pulsing between 0 and 2 V for 16,000 cycles with a pulse length of 0.02 s in 0.5 M H_2_SO_4_ to increase the microscopic surface area of the electrodes. We then immersed the electrodes in 0.5 M H_2_SO_4_ and cycled the potential two times between 1.50 and −0.35 V at 100 mV/s. Finally, we rinsed the freshly cleaned electrodes with deionized water.

We modified the gold electrodes as follows [62]. First, we combined 6 µL of 10 mM Tris (2‐carboxyethyl) phosphine (TCEP) per microliter of the DNA‐aptamer at 100 µM to reduce DNA constructs, which the manufacturer provides in a disulfide form. The solution is incubated for 1 h in the dark at room temperature. We prepared 10 mM MCH by dissolving 4.05 µL pure MCH in 3 mL PBS and then diluted to 100 µM using PBS. We combined 10 µL of 100 µM MCH per 3.5 µL reduced DNA solution and then diluted them to 100 µL in PBS to get a mixture of 500 nM DNA and 10 µM MCH. Finally, we prepared sensors by immersing clean working electrodes in the prepared mixture overnight. We rinsed all sensors with deionized water prior to measurements.

### 4.3 Sensor Interrogation

To interrogate our sensors, we performed square wave voltammetry at frequencies of 15 and 100 Hz, 25 mV amplitude, potential range −0.20 to −0.45 V (all potentials relative to Ag/AgCl). This was performed on a CHI 1040C Electrochemical Workstation.

### 4.4 Calibration

Historically, we have calibrated new EAB sensors by titrating them against their target in vitro in the biological matrix in which they will be deployed [6]. Here, we held sensors in fresh and heparinized rat blood at 37°C. Following this, we then perform a single‐point calibration at a known target concentration (typically zero if the target is exogenous and the animal had not yet been dosed). In the case of tryptophan, two complications render calibration more complex.

The first complication is that endogenous tryptophan concentrations vary from animal to animal, precluding the easy calibration of each sensor in vivo prior to first challenging the animal with exogenous tryptophan. To circumvent this, we have employed a recently described calibration‐free approach to EAB sensor operation, termed ratiometric kinetic differential measurements (rKDM), which avoids the need to calibrate each individual sensors [38]. Specifically, to produce the calibration curve, we extracted the voltammetric peak current of 15 and 100 Hz frequency at each target concentration (after at least 3 min equilibration to ensure the proper temperature had been achieved). We converted these into a calibration‐free kinetic differential measurement value, rKDM, with values observed at the two frequencies (*i*_1_ and *i*_2_) using the following Equation [38]:

(10) $$rKDM=\frac{i_{2}-Ri_{1}\;}{0.5\left(i_{2}+Ri_{1}\right)}$$

where *R* is the ratio of the currents measured in the absence of target at 100 Hz, *i_2_*, to that measured in the absence of target seen at 15 Hz, *i_1_*. In blood at 37°C *R* = 2.1 for the sensors employed here. We fit the resulting rKDM values to a Langmuir isotherm to correlate them with the measured target concentration:

(11) $$rKDM=\frac{\left[T\right]\Delta S}{\left[T\right]+K_{D}}\;$$

where [T] is the target concentration, Δ*S* is the rKDM signal change seen at saturated free target, and *K_D_* is the aptamer's dissociation constant. The target concentration is then determined from in vivo measurements as:

(12) $$\left[T\right]=K_{D}\frac{rKDM}{\Delta S-rKDM}$$

The second complication regarding the calibration of tryptophan measurements is that, at physiological concentrations, only 10%–20% of the amino acid in circulation in blood is free in solution, with the rest being protein‐bound [63]. EAB sensors, however, respond only to free (unbound) target, which is also, presumably, the physiologically relevant form of the metabolite (e.g., available to enhance the lifetime of TDO). To account for this, we titrated our EAB sensor in fresh rat blood to which we added increasing concentrations of exogenous tryptophan. At each step in the titration, we removed an aliquot of the blood sample. We centrifuged these blood samples to remove blood cells, and their supernatants were then spun through a 10 kDa molecular‐weight cut‐off filter to remove plasma proteins. The resulting filtrate was analysed to determine free tryptophan using a commercial assay kit (Merck Millipore, Billerica, MA). We then fit the measured free tryptophan to a Langmuir isotherm, producing the equation we used to convert EAB signal to concentration estimates in our in vivo studies (Figure 2A).

### 4.5 In Vivo Measurements

We employed 4‐ to 5‐month‐old Sprague–Dawley rats (Charles River Laboratories of Wilmington, MA) for our in vivo studies. The rats, weighing between 300 and 650 g, were pair‐housed in a reverse light cycle room (12:12 regular light cycle with lights off at 10 AM), with ad libitum access to food and water. Our experimental protocol, approved by the Institutional Animal Care and Use Committee (IACUC) of the University of California at Santa Barbara, adhered to the guidelines outlined in the NIH Guide for Care and Use of Laboratory Animals (eighth edition, National Academy Press, 2011).

For sensor placement, the rats were anesthetized using 4% isoflurane in a Plexiglas anaesthesia chamber, and anaesthesia was maintained via a nose cone throughout the experiment at 2%–2.5% isoflurane. Bilateral incisions were made on the neck to surgically isolate the left and right jugular veins. Each vein was tied off using sterile 6–0 silk sutures (Fine Science Tools, Foster City, CA). Spring‐loaded microscissors were then used to make a small incision in each vein, allowing insertion of the sensor‐containing catheter into the right jugular vein and an infusion line into the left jugular vein. Both the sensor and drug infusion catheter were anchored in place using two sterile 6–0 silk sutures. Prior to measurement, the wires in the in vivo sensor were adjusted to expose the counter and working electrode outside of the 20G catheter, and 30 units of heparin were infused through the infusion line immediately after insertion of the sensor and prior to any recordings.

While inserting the sensor into the vein may cause local vascular perturbation, under the acute measurement conditions used here we expect that implantation‐related effects primarily influence sensor stability rather than dominate systemic plasma tryptophan dynamics. Consistent with this, the tryptophan *C_max_* and subsequent systemic clearance rates we observe are consistent with the results of prior, blood‐draw‐derived studies [30, 31, 32, 33].

In vivo measurements were collected on isoflurane‐anesthetized animals using the following protocols. For intravenous tryptophan challenges, the appropriate volume of 40 mM tryptophan solution (in PBS solution) was injected into the left jugular vein over 1 min using a syringe pump (KD Scientific). For LM10 dosing, the appropriate volume of 25 mM LM10 in DMSO was injected intraperitoneally using a syringe. The electrical signal of in vivo sensors in the rats was recorded throughout the experiments and then converted into free tryptophan concentrations to obtain the tryptophan kinetics (Figures 3, 4, 5). These kinetics were fitted with a first‐order model or a cooperative Michaelis‐Menten model using equations 1 and 2 in MATLAB. During the recorded time of the experiments, we collected blood samples from 2 rats to perform ex vivo measurements of the free plasma tryptophan concentration for comparison with our EAB sensor data. Blood samples were collected from the jugular vein via the same catheter used for tryptophan administration. Because rats have a limited total blood volume, repeated or excessive blood sampling can perturb physiological function and compromise animal welfare. We therefore limited blood collection to two samples per animal: one before administration of the tryptophan challenge and one after. Collected blood samples were treated to obtain free tryptophan concentrations using the same method we employed to derive our calibration curve.

## Author Contributions

**Zeki Duman**: software, data curation, formal analysis, visualization. **Yuyang Wu**: conceptualization, methodology, software, data curation, validation, investigation, visualization, writing – review and editing, writing – original draft, formal analysis. **Rose Mery Bakestani**: data curation. **Carl M. Kirkpatrick**: conceptualization, methodology. **Joao Hespanha**: software, formal analysis, visualization. **Tod Kippin**: methodology, conceptualization, supervision, funding acquisition, resources. **Chelsea N. Brown**: investigation, methodology, validation, data curation. **Kevin W. Plaxco**: supervision, funding acquisition, writing – original draft, writing – review and editing, methodology, conceptualization, resources, project administration.

## Conflicts of Interest

The authors declare no conflicts of interest.

## Supporting information




**Supporting File**: advs77595‐sup‐0001‐SuppMat.docx.

## Data Availability

The data that support the findings of this study are available in the supplementary material of this article.
