# Supplementary material for: Highly‐Time‐Resolved Measurements of Tryptophan's Distribution and Homeostatic Regulation in Live, Intact Animals
Source: Adv Sci (Weinh). 2026 Oct 15:e77595. Online ahead of print. doi: 10.1002/advs.77595 (PMC13672249; doi:10.1002/advs.77595)
Supplement: Supplementary file 1 [file ADVS-9999-e77595-s001.docx]

**Supporting Information for**

**Highly-time-resolved measurements of tryptophan’s distribution**

**and homeostatic regulation in live, intact animals**

Yuyang Wu^1^, Chelsea N. Brown^2^, Zeki Duman^3^, Rose Mery Bakestani^1^, Carl M. Kirkpatrick^4^,

Joao Hespanha^3^, Tod Kippin^5,6,7^, and Kevin W. Plaxco^1,7*^

^1^Department of Chemistry and Biochemistry, University of California Santa Barbara, Santa Barbara, CA 93106, USA

^2^Institute of Collaborative Biotechnology, University of California Santa Barbara, Santa Barbara, CA 93106, USA

^3^Department of Electrical and Computer Engineering, University of California Santa Barbara, Santa Barbara, CA 93106, USA

^4^Monash Institute of Pharmaceutical Sciences, Monash University, Melbourne, Australia

^5^Department of Psychological and Brain Sciences, University of California Santa Barbara, Santa Barbara, CA 93106, USA

^6^Neuroscience Research Institute, University of California Santa Barbara, CA 93106, USA

^7^Biological Engineering Graduate Program, University of California Santa Barbara, Santa Barbara, CA 93106, USA

*Corresponding author: kwp@ucsb.edu

**Figure SI1.** Shown are the fit residuals of the first-order and cooperative Michaelis-Menten models immediately after peak concentrations are reached for the 100 mg/kg tryptophan challenge (Fig. 2). Over the first 30 min, the root-mean-squared deviation for the two are 16 µM and 9 µM, respectively.

**Figure SI2.** Shown are the TDO activity “zoom-ins” version of Figure 5. Note that the TDO activity axes (the right-hand y-axes) no longer start at zero.

**Figure SI3.** By BIC, the variable-TDO model is as equally favored as the fixed-TDO model for our (A) dual challenge data set (BIC = 1884 and 1883, respectively). In contrast, however, the former model is the strongly preferred description of our (B) triple (5408 versus 5447) and (C) quadruple (5711 versus 5728) challenge data sets. Zoom-in TDO activity changes are the same as those in Figure SI2.

**Figure SI4.** Shown are the “zoom-ins” of the change in basal free plasma tryptophan concentrations after dosing with 10 mg/kg LM10. These data are from Fig. 4A (Black curve), 4B (Blue curve) and 4C (Orange curve).

**Figure SI5.** Shown are the TDO activity “zoom-ins” version of Figure 6. Note that the TDO activity axes (the right-hand y-axes) no longer start at zero.

**Figure SI6.** BIC indicates that one-compartment model is the favored descriptor of challenge data collected in the absence of LM10. Zoom-in TDO activity changes are the same as those in Figure SI2.

**Figure SI7.** The one-compartment model we employed to fit most of our data does not accurately capture the return to homeostasis kinetics seen for tryptophan challenges delivered after LM10 dosing. These data are, however much better descried by a two-compartmental model, perhaps because this TDO inhibitor also inhibits the transport of tryptophan into cells. Denoted below each graph are fitted plasma rate constants for the 2-compartment model. To note, these data are the same dataset from fig 4 but subtracted after the initial tryptophan plasma level reaches equilibrium following LM10 dosing.
